# Combating Salinity Through Natural Plant Extracts Based Biostimulants: A Review

**DOI:** 10.3389/fpls.2022.862034

**Published:** 2022-05-20

**Authors:** Ali Ahmad, Begoña Blasco, Vanessa Martos

**Affiliations:** ^1^Department of Plant Physiology, University of Granada, Granada, Spain; ^2^Institute of Biotechnology, University of Granada, Granada, Spain

**Keywords:** salt stress, stress perception, signaling signatures, NaCl, bioactive compounds, climate change, antioxidants, osmoprotectants

## Abstract

Enhanced crop growth and yield are the recurring concerns in agricultural field, considering the soaring world population and climate change. Abiotic stresses are one of the major limiting factors for constraining crop production, for several economically important horticultural crops, and contribute to almost 70% of yield gap. Salt stress is one of these unsought abiotic stresses that has become a consistent problem in agriculture over the past few years. Salinity further induces ionic, osmotic, and oxidative stress that result in various metabolic perturbations (including the generation of reactive oxygen, carbonyl, and nitrogen species), reduction in water potential (ψ_w_), distorted membrane potential, membrane injury, altered rates of photosynthesis, leaf senescence, and reduced nitrogen assimilation, among others); thereby provoking a drastic reduction in crop growth and yield. One of the strategies to mitigate salt stress is the use of natural plant extracts (PEs) instead of chemical fertilizers, thus limiting water, soil, and environmental pollution. PEs mainly consist of seeds, roots, shoots, fruits, flowers, and leaves concentrates employed either individually or in mixtures. Since PEs are usually rich in bioactive compounds (e.g., carotenoids, flavonoids, phenolics, etc.), therefore they are effective in regulating redox metabolism, thereby promoting plant growth and yield. However, various factors like plant growth stage, doses applied, application method, soil, and environmental conditions may greatly influence their impact on plants. PEs have been reported to enhance salt tolerance in plants primarily through modulation of signaling signatures and pathways (e.g., Na^+^, ANNA4, GIPC, SOS3, and SCaBP8 Ca^2+^ sensors, etc.), and regulation of redox machinery [e.g., superoxide dismutase (SOD), catalase (CAT), ascorbate peroxidase (APX), non-specific peroxidase (POX), glutathione peroxidase (GPX), peroxiredoxin (Prx), ascorbic acid (AsA), glutathione (GSH), α-tocopherol, etc.]. The current study highlights the role of PEs in terms of their sources, methods of preparation, and mode of action with subsequent physiological changes induced in plants against salinity. However, an explicit mode of action of PEs remains nebulous, which might be explicated utilizing transcriptomics, proteomics, metabolomics, and bioinformatics approaches. Being ecological and economical, PEs might pave the way for ensuring the food security in this challenging era of climate change.

## Introduction

The most defining concern of the present and future agriculture, and of humanity, is climate change. Human dependence on wild grains began in Halocene—a geological epoch, which further laid the basis of agriculture, as it was characterized by stable and warm temperatures. However, a rapid surge in the atmospheric carbon dioxide levels from 100 ppm to over 400 ppm with an average temperature increase by 1°C, in the past 70 years, could be a steppingstone for unstable environmental temperatures. A further escalation of temperature by 3°C by the year 2100, and even by 8°C or more is also predicted. Such drastic escalations have been identified as antagonists of human civilization (Gowdy, 2020). Similarly, agricultural activities, particularly, the use of chemical fertilizers not only incites diabetes and cancer like chronic diseases in humans, but also deteriorates the environment; thereby aggravating the climate change (Ahmad et al., 2021b). In this regard, European Union (EU) has implemented a “European Green Deal” program that proposes a 20% reduction in the use of fertilizers and a 55% reduction in greenhouse gases by 2030 as compared to 1990 levels (European-Union, 2020). Apart from this, soaring human population that is expected to reach 9.7 billion by 2050, is amplifying the pressure on agriculture to meet the growing food demands globally. Substantially lower crop yield ha^–1^ in comparison to increasing world population is already reported by World Food Program (WFP). Likewise, by the year 2100 a decrease in the yield of maize, wheat, and rice, by 20–45, 5–50, and 5–50%, respectively, has been predicted by Food and Agriculture Organization (FAO) if the climate change remains incessant (Arora, 2019). This ever-increasing food demand has provoked intensive agriculture systems with the unprecedented use of chemical fertilizers, pesticides, herbicides, fungicides, along with the exploitation of land and water resources; thereby further aggravating the climate change. If such trends persist, they will not only compromise the global food safety and security but may also provoke a civilization collapse.

Climate change provokes a plethora of abiotic stresses (flooding, drought, salinity, etc.) in plants (Arora, 2019; Gowdy, 2020). These abiotic stresses are one of the major limiting factors for constraining crop production (Ramadoss et al., 2013), for several economically important horticultural crops, and contribute to almost 70% of yield gap (Rouphael and Colla, 2020). Salinity is one such abiotic stress that hampers plant physiological functioning in a number of ways: resulting in lower crop production. It is attributed as a measure of salt amount in soil or water content (Arif et al., 2020), and is classified as primary salinity: caused as a result of natural processes i.e., rain, weathering, wind, etc., and secondary salinity: caused as a result of anthropogenic activity, i.e., excessive irrigation, deforestation, clearing land (Munns and Tester, 2008; Arif et al., 2020). The climate change is directly responsible for primary salinity considering the excessive rains only, for example 100 mm of rainfall year^–1^ would deposit 10 Kg ha^–1^ of sodium chloride if it contains 10 mg Kg^–1^ of salt (Munns and Tester, 2008), whereas indirectly to secondary salinity. Subsequently, intensive agricultural systems and poor agronomic practices, like over-fertilization, desertification, excessive irrigation, etc., have increased the levels of alkalinity and salinity of cultivated soils (Arora, 2019; Li et al., 2021). In 2013, the salinity affected cultivated soils were estimated to be over 800 million ha (Ramadoss et al., 2013) globally that culminated to around 900 million ha in 2020 (Velmurugan et al., 2020). Such a rapid conversion of fertile soils into saline ones can be regarded as a major threat to food security and agricultural sustainability.

Salinity is expressed as the electric conductivity (EC) of the soil solution, and the soil is generally denominated as saline if its EC is 4 dS m^–1^ or more, it approximately equals to 40 mM NaCl, producing a 0.2 MPa approximate osmotic pressure (Munns and Tester, 2008). Sodium ions (Na^+^) are considered as major contributors to salinity, whereas Cl^–^, Mg^2+^, SO_4_^2–^, or HCO_3_^–^ are also responsible for soil salinity but to a lesser extent (Munns and Tester, 2008; Zörb et al., 2019). Higher concentrations of these salts trigger ionic and osmotic stress resulting in the generation of reactive oxygen species (ROS), reduced cell/leaf expansion, leaf abscission, reduction in ψ_w_, distorted membrane potential, membrane injury, altered rates of photosynthesis, stomatal closure, protein destabilization, altered carbon portioning, cavitation, reduced nitrogen assimilation, ion cytotoxicity, and cell death among others; thereby provoking a drastic reduction in crop growth and yield (Ashraf and Harris, 2004; Munns and Tester, 2008; Acosta-Motos et al., 2017; Farooq et al., 2017; Arif et al., 2020). Therefore, it is indispensable to devise novel strategies for combating salinity.

Use of natural plant extracts (PEs) (or “botanicals”) could be one of the salinity mitigation ecofriendly strategies. PEs are potential alternatives to chemical fertilizers. PEs fall under the umbrella of plant biostimulants, and are used to enhance plant growth (Calvo et al., 2014; Drobek et al., 2019). PEs are the concentrates of plants and could be prepared using any part of the plant, i.e., seeds, roots, stems, leaves, bark, flowers, etc. (Semida and Rady, 2014; Lorenzo et al., 2019; Nessim and Kasim, 2019; Rady et al., 2019b; Zulfiqar et al., 2020b). The application of PEs could be either in liquid form as foliar spray and/or root treatment, or as soil preparations like granules, concentrates, solutions added to soil, or powders (Drobek et al., 2019). PEs can be associated with the amelioration of salinity as they are the sources of prominent phytochemicals like vitamins, carotenoids, amino acids, phytohormones, mineral nutrients, phenolics, and antioxidants (Latef et al., 2017). There are several reports where these compounds, used either individually or in mixtures, were found to be effective against salinity (Calvo et al., 2014; Iqbal et al., 2014; Bulgari et al., 2015; Drobek et al., 2019; Shukla et al., 2019; Zulfiqar et al., 2020b). However, the effect of PEs is often concentration dependent. Similarly, plant part and age of plant used as an extract also influences the PEs overall proficiency.

Currently, there are no extensive studies reported on the particular use of PEs and their subsequent mechanism of action against salinity. Previously reported studies mostly discuss the use of biostimulants that is rather a broader term and even includes microbial and non-microbial formulations, protein hydrolyzates, PEs, amino acid, seaweed extracts, etc. (Du Jardin, 2015). Furthermore, previous studies have discussed the use of biostimulants on abiotic stresses in general (Calvo et al., 2014; Drobek et al., 2019; Shukla et al., 2019; Rouphael and Colla, 2020; Zulfiqar et al., 2020b), whereas no specific study on the use of PEs against salinity is reported. Therefore, the aim of the study was to elaborate the potential of ecofriendly and natural PEs as salinity alleviators, and to underline their possible mode of action with the subsequent physiological changes thus induced. Since an explicit mode of action of PEs remains nebulous, hence this subject has been estimated considering the up or down regulation of signaling signatures, altered photosynthetic rates, and redox metabolism in general.

This review is divided into three sections. Impact of plant based biostimulants under normal conditions is discussed first followed by salinity induced physiological, biochemical, and genetic changes in plants. Subsequently, the use of PEs, including their sources and methods of preparation, as salinity mitigation strategy is discussed. Finally, all this is concluded by identifying the limitations and future perspectives of the use of PEs against salinity.

## Plant Based Biostimulants

Use of plant based biostimulants is rapidly gaining popularity in agriculture. Plant based biostimulants, apart from inducing stress tolerance, are also effective in regulating a number of plants physiological processes; thereby improving plant growth and yield (Brown and Saa, 2015). They may comprise of protein hydrolyzates and amino acids, hormone-, amino acids-, or nutrients containing products, vegetable oils, etc. of plant origin (Ikrina and Kolbin, 2004; Kauffman et al., 2007; La Torre et al., 2016; Yakhin et al., 2017). Their mechanism of action might involve phosphorus (P) release from soils, activation of nitrogen (N) metabolism, stimulation of root growth, nutritional and hormonal regulation, or generic stimulation of soil microbial activity. Previous reports have documented that the application of biostimulants has enhanced various physiological processes including plant nutrient uptake and utilization, photosynthesis, water use efficiency, synthesis and concentration of growth hormones (auxins, gibberellins, and cytokinins), germination, and senescence reduction (Parađiković et al., 2011; Bulgari et al., 2015; Nasir et al., 2016; Merwad, 2017; Ur Rehman et al., 2017; Milić et al., 2018; Younis et al., 2018), which in return increase plant production, yield, post-harvest quality, and shelf life of agricultural products.

Among various plant based biostimulants, PEs are economical and easy to prepare. Several studies have reported their beneficial effects on plants growth and yield. For instances, an increase in the growth and hormonal profile was observed in eggplant and snap bean when aqueous garlic extracts were applied (Elzaawely et al., 2018; Ali et al., 2019). Similar results were reported in case of moringa leaf extracts being applied on sword lily, where they improved plant growth and vase life by regulating various physiological processes (Zulfiqar et al., 2020c). Likewise, borage extracts were reported to supplement the primary metabolism, by enhancing leaf pigments and photosynthetic activity, and reduced chlorophyl a fluorescence, by incrementing the number of active reaction centers per cross section, in lettuce plants (Bulgari et al., 2017). Likewise, vine-shoot and oak extracts were found to significantly improve wine yield and quality by triggering amino acids and volatile compounds production (Sánchez-Gómez et al., 2016). In addition, PEs are also responsible for increasing the shelf life and postharvest quality of agricultural products. For example, moringa leaf extracts were found to significantly improve avocado and citrus fruit shelf life and quality by lowering the respiration rate and water transfer from the fruits (Adetunji et al., 2012; Tesfay and Magwaza, 2017). All of these studies indicate the potential of PEs in positively regulating several physiological processes. Therefore, extrapolating the incredible potential of PEs to combat abiotic stresses, especially salinity, would be a promising approach for a sustainable agriculture.

## Salinity Perception and Signaling in Plants

**FIGURE 1:**
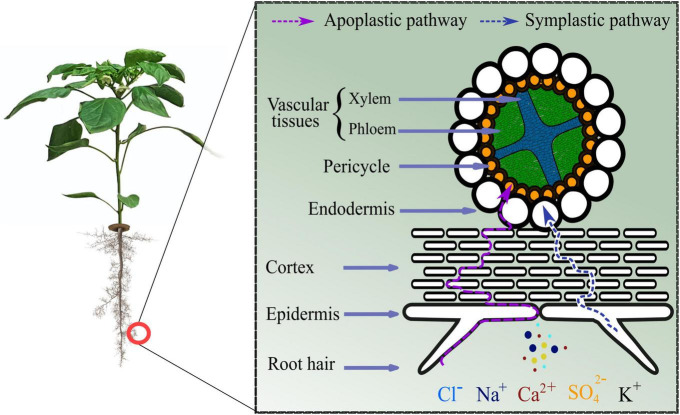
Entry routes for salinity causing ions.

**FIGURE 2:**
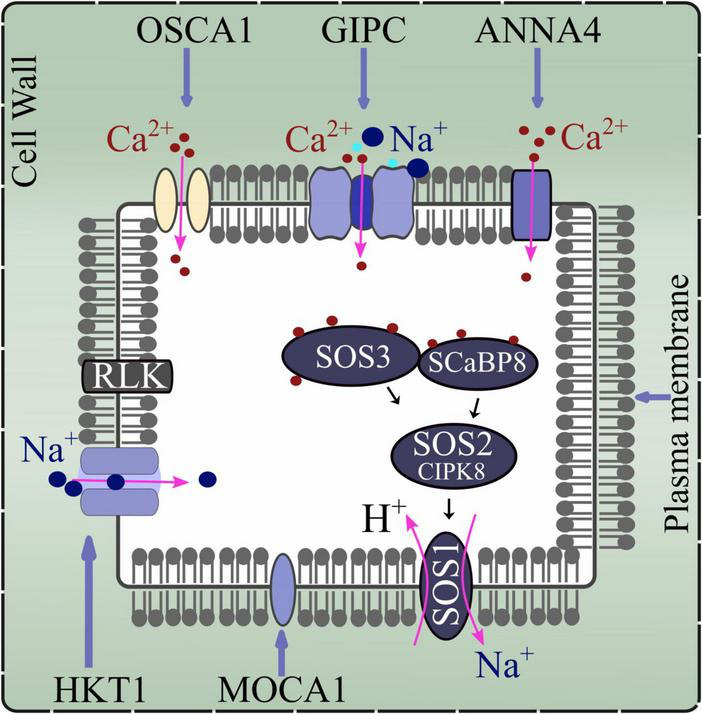
Representation of membrane-based proteins, and ion channels that regulate ions movement in plants (under saline environment).

Stress perception and signaling hold an imperative role for subsequent plant behavior. Apoplastic and symplastic pathways are the recognized routes of ions entry into plant that result in salinity (Lamers et al., 2020) (Figure 1). Stress signals are perceived by plant cell surface-based receptors that stimulate the production of secondary messengers like Ca^2+^, ROS, and inositol phosphates, among others (Rao et al., 2016). The most common surface based receptors involved in the cation sensing (Na^+^ and Ca^2+^) are membrane bound proteins or ion channels including glycosyl inositol phosphorylceramide (GIPC) sphingolipids synthesized by MONOCATION-INDUCED [Ca^2+^]_cyt_ INCREASES1 (*MOCA1*), *Arabidopsis* ANNEXIN4 (*ANN4*), and *Arabidopsis* HIGH-AFFINITY K^+^ TRANSPORTER1 (*HKT1*) (Chen et al., 2021). Similarly, ethylene receptors like *Nicotiana tabacum* histidine kinase 1 (*NTHK1*) are also reported to modulate stress signaling (Cao et al., 2008). While Ca^2+^ signaling is an important mechanism for salt-sensing and is modulated through SALT OVERLY SENSITIVE (SOS) pathway. SOS pathway consists of SOS1 Na^+^/H^+^ antiporter, SOS2 and SOS2-LIKE PROTEIN KINASE5 (PKB5) protein kinases, and SOS3 and SCaBP8 Ca^2+^ sensors (Lamers et al., 2020). The intercellular Ca^2+^ perturbations trigger Ca^2+^ sensors, e.g., SOS3 and SCaBP8, bringing about conformational changes in them in a calcium-dependent manner. These sensors further interact with their respective partners, e.g., activate SOS2, whereby a phosphorylation cascade is initiated (Quan et al., 2007; Rao et al., 2016). Subsequently, SOS1 is activated carrying out the efflux of Na^+^ ions (Figure 2). It is reported that within 20 s of stress (sodium) application a change in SOS1 exchanger activity can be detected (Lamers et al., 2020). Ultimately these alterations result in the genomic regulations (activation of transcription factors and stress responsive genes) by biosynthesizing metabolites and other compounds needed to combat salinity. There are two categories of stress- responsive genes, i.e., (a) early induced genes: immediate activation on receiving the stress signal with short-term persistence (including transcription factors, interfering RNAs, etc.), and (b) late induced genes: late activation with longer persistence periods (including membrane stabilizing, osmolytes, antioxidants, etc.) (Rao et al., 2016).

Since plants undergo an osmotic stress under saline environment, therefore it is proposed that receptors involved in osmotic, or drought sensing can also be implicated for salinity signal transduction. It is reported that a plasma membrane located channel encoded by *OSCA1* regulates the Ca^2+^ influx under plasma membrane tension or extracellular osmotic pressure. Similarly, the receptor-like kinases (RLKs) present on plasma membrane have also been reported to play certain role (Lamers et al., 2020) (Figure 2).

## Plant Responses to Salinity

Plants generally vary in their response to salinity. Their response could be cellular- and tissue level, morphological, or physiological. Such response depends on various factors including duration and severity of stress, plant age and its developmental stage, and plant species (Peleg et al., 2011). Therefore, some plants are found to be more tolerant (less sensitive) to salinity than others. For example, barley (*Hordeum vulgare*) is more tolerant to salinity than rice (*Oryza sativa*) (Munns and Tester, 2008). Plants are generally categorized as halophytes or euhalophytes based on their genetic adaptability to salinity, whereas they are termed as glycophytes if they are less-tolerant or not adapted to salt stress (Acosta-Motos et al., 2017). The general effects of salt stress in glycophytes occur in the following two forms (Munns and Tester, 2008; Acosta-Motos et al., 2017);

(i) Immediate response: onset of osmotic stress on surpassing the threshold limit of salts in the root zone resulting in the reduction of shoot biomass.
(ii) Slower response: onset of ionic stress on accumulation of salt ions (Na^+^ and Cl^–^) in older leaves resulting in the impairment of photosynthetic machinery and leaf senescence.

The rapid osmotic phase response of plants begins at the root-soil periphery. The toxic concentrations of salt ions build up osmotic pressure that negatively regulates the rate of leaf expansion, emergence of new leaves, lateral buds- and shoots formation. The second phase is characterized by the higher accumulation rate (i.e., toxic concentrations) of Na^+^ ions in the older leaves that cannot dilute these salts due to lack of expansion, ultimately resulting in their death. Although, some plant species are sensitive to higher Cl^–^ concentrations. This results in the reduced photosynthetic rates of plants that causes a reduction in their growth rate. Since ionic stress is time taking due to the accumulation of ions, therefore plant growth is affected much later with lesser impact as compared to osmotic stress.

## Mechanisms of Salinity Tolerance in Plants

Salinity tolerance in plants is achieved through a series of complex signaling and biosynthetic responses. However, the commonly known mechanisms of salinity tolerance include morphological (roots and aerial parts), metabolic (osmotic regulation, ionic and molecular homeostasis, and hormonal homeostasis), and genetic responses. Comprehensive reports of salinity tolerance mechanisms have been reported previously (Munns and Tester, 2008; Gupta and Huang, 2014; Tang et al., 2015; Acosta-Motos et al., 2017; Farooq et al., 2017; Arif et al., 2020). Here, a description of these mechanisms is presented to elaborate the use of PEs, as they reinforce these salinity tolerance mechanisms.

### Morphological Adjustments

Plants can adapt their morphological features to sustain the normal functioning and cellular homeostasis in case of any unfavorable stimulus. This is characterized as phenotypic plasticity. This also occurs in case of salinity. Although, it varies among salt tolerant and salt sensitive species. Generally, the productivity or yield of an agricultural crop can be assessed by analyzing its above-and below-ground parts. Therefore, few of the growth indices, as per Beadle (1993), taken into consideration for salt stress studies in plants are listed below:


R⁢e⁢l⁢a⁢t⁢i⁢v⁢e⁢g⁢r⁢o⁢w⁢t⁢h⁢r⁢a⁢t⁢e⁢(R⁢G⁢R)= ⁢(l⁢n⁢W⁢2/W⁢1)/(t⁢2-t⁢1)

N⁢e⁢t⁢a⁢s⁢s⁢i⁢m⁢i⁢l⁢a⁢t⁢i⁢o⁢n⁢r⁢a⁢t⁢e⁢(N⁢A⁢R)= ⁢[(W⁢2-W⁢1)/(A⁢2-A⁢1)]⁢[(l⁢n⁢A⁢2/A⁢1)/(t⁢2-t⁢1)]

L⁢e⁢a⁢f⁢a⁢r⁢e⁢a⁢r⁢a⁢t⁢i⁢o⁢(L⁢A⁢R)= ⁢[(A⁢2-A⁢1)/(W⁢2-W⁢1)]⁢[(l⁢n⁢W⁢2/W⁢1)/(l⁢n⁢A⁢2/A⁢1)]

S⁢p⁢e⁢c⁢i⁢f⁢i⁢c⁢l⁢e⁢a⁢f⁢a⁢r⁢e⁢a⁢(S⁢L⁢A)= ⁢[(A⁢2-A⁢1)/(W⁢L⁢2-W⁢L⁢1)]⁢[(l⁢n⁢W⁢L⁢2/W⁢L⁢1)/(l⁢n⁢A⁢2/A⁢1)]

L⁢e⁢a⁢f⁢w⁢e⁢i⁢g⁢h⁢t⁢r⁢a⁢t⁢i⁢o⁢(L⁢W⁢R)= ⁢[(W⁢L⁢2-W⁢L⁢1)/(W⁢2-W⁢1)]⁢[(l⁢n⁢W⁢2/W⁢1)/(l⁢n⁢W⁢L⁢2/W⁢L⁢1)]

Where W represents the total dry weight, WL is the total dry weight of leaves, A is the total leaf area, t is the time, 1 and 2 represents the start and end of a period, respectively. Considering these growth indices as standard, various studies have demonstrated relative effects of salinity on plant morphology as a decrease in RGR in five ornamental plants (Cassaniti et al., 2012), a decrease in NAR in *Hibiscus cannabinus* and *Argyranthemum coronopifolium* (Curtis and Lauchli, 1986; Morales et al., 1998), a decrease in LAR in *A. coronopifolium* (Morales et al., 1998), and an increase in LWR in *Asteriscus maritimus* (Rodrıguez et al., 2005). Similarly, other morphological changes observed in plants (salt sensitive) under salinity include decrease in leaf thickness, succulence values, surface to volume ratio of cells and tissue density, spongy parenchyma, and number of mitochondrial cristae, whereas an increase in the lower area/volume ratio of mesophyll cells, mesophyll thickness, leaf water balance, leaf size, palisade cell size, succulence values, intercellular space, and palisade parenchyma has been reported (Longstreth and Nobel, 1979; Romero-Aranda et al., 1998; Franco-Navarro et al., 2016; Acosta-Motos et al., 2017). Most of these changes suggest that plants under salt stress intend to increase the CO_2_ diffusion so that the energy production should not be disrupted along with an increased water use efficiency through higher photosynthetic performance. Similarly, leaf senescence and leaf color change are also salinity mitigation mechanisms, in which chlorophyll is gradually degraded resulting in the accumulation of carotenoids and anthocyanins that provide protection against oxidative stress (Hörtensteiner, 2006; Garriga et al., 2014).

Plant roots experience some morphological changes in their size, diameter, and number so as to maximize the nutrients and water uptake. Increased root to shoot ratio helps plants in compartmentalization and ions retention. Likewise, root proliferation helps plant to curb toxic ions accumulations. Also, root density and electrical conductivity increases under saline environment (Acosta-Motos et al., 2017; Arif et al., 2020). The other anatomical and ultra-structural change under salinity is the development of casparian strip and suberin lamella serving as apoplastic barrier. Similarly, plants’ chloroplast number and stable size structure increases under salinity (Arif et al., 2020) to maintain the effective photosynthetic rates.

### Metabolic Adjustments

Salinity creates an ionic imbalance in plants that may result in denaturation or damage to subcellular organelles like chloroplasts and mitochondria. Therefore, plants compartmentalize the excessive ions into their vacuoles, and usually put into play their inclusion and exclusion mechanisms. This is generally regulated by (a) sequestration of excessive salt into vacuole with the help of various pumps (e.g., Na^+^/H^+^ antiporters); (b) ionic equilibrium: modulation of Na^+^, Cl^–^, K^+^, and Ca^2+^ in the plant cell via SOS and non-selective cationic channels (NSCCs) (Gupta and Huang, 2014; Arif et al., 2020). Osmotic potential (ψ_s_) of plant cell is critical for its growth, development, and yield. That is why its proper regulation is important. Plants generally produce osmoprotectants to keep in check their ψ_s_. These compounds are also described as compatible solutes. Unlike ions they neither paralyze the metabolic functions of enzymes, nor they destabilize the cellular membranes. In comparison to inorganic compounds, higher concentrations of these compounds are non-toxic to cellular metabolism (Nahar et al., 2016). Additionally, osmoprotectants play a diverse role in plant physiology under harsh conditions. Some of their prominent roles regarding metabolic adjustments in plants under stress include stabilization of proteins structures, regulation of protein folding, detoxification of ROS, stabilization of thylakoid membranes, protection of antioxidant enzymes, regulation of redox balance, and activation of stress responsive genes that result in redox homeostasis, stress signaling, upregulation of photosynthesis, and scavenging of toxic radicals (Zulfiqar et al., 2020a). These compatible solutes include betaines [proline betaine, hydroxyproline betaine, glycine betaine (GB), and pipecolate betaine], proline, sugars (fructose, glucose, sucrose, and fructans), and sugar alcohols (mannitol, sorbitol, and inositol). Of these compatible solutes, GB, a quaternary ammonium compound, usually ameliorates the toxic effects by accumulating in the cell and by distinguishing Na^+^ to K^+^ ratio. It is also reported to safeguard PSII under salt stress. Also, proline acts as an osmoprotectant as well as a molecular chaperone sustaining the structural integrity of macromolecules. Similarly, higher amounts of reduced sugars, i.e., fructose, glucose, sucrose, and fructans, stabilize the membrane integrity and prevent them from denaturation. Likewise, mannitol, sorbitol, and inositol facilitate in maintaining turgor, Na^+^ sequestration into the vacuole, and quenching ROS (Tang et al., 2015; Acosta-Motos et al., 2017; Arif et al., 2020).

One of the most common abiotic stress indicators in plants is the induction of oxidative stress. Salt stress also results in oxidative stress that comprises of ROS, reactive carbonyl species (RCS), and reactive nitrogen species (RNS) (Mano, 2012; Corpas, 2016; Fancy et al., 2017). However, these indicators of stress are also found in plant cells under normal conditions and a proper regulation of their intrinsic cellular concentration exists because they are also involved in plant growth and development, and signaling at subcellular and intercellular level (Corpas, 2016; Fancy et al., 2017; Zulfiqar and Ashraf, 2021a). Under any unfavorable condition, homeostatic balance of these reactive species disrupts resulting in altered cellular redox potential that results in the denaturation of various vital compounds including nucleic acids, proteins, lipids, etc. and disruption of cellular structures (Mano, 2012; Hasanuzzaman et al., 2020; Zulfiqar et al., 2020a). Nitric oxide (NO) and derived molecules altogether constitute RNS, whereas methylglyoxal (MG) and other α,β-unsaturated carbonyl compounds constitute RCS that are more stable than ROS (Mano et al., 2019; Nareshkumar et al., 2020). Mitigation of these radicals protect plant organelles in a number of ways. For example, the photoproduction and removal of ROS not only protects the chloroplast from the damaging effects of ROS but also acts as an escape valve for excess photons (Hasanuzzaman et al., 2020). Similarly, MG detoxification may result in improved cell proliferation, miotic index, seed germination, photosynthesis, stress-related gene expression, etc. (Mostofa et al., 2018). Preferred sites of ROS generation have been identified as chloroplast, mitochondria, and peroxisomes, whereas for NO as peroxisomes—although this remains a subject of further research (Hernandez et al., 1995; Hernández et al., 2001; Corpas, 2016). Similarly, RCS are generated as a by-product in various metabolic pathways, i.e., sugar metabolism, oxidative degradation of glucose and glycated proteins, glycolysis, lipid peroxidation, photosynthesis, etc., (Kaur et al., 2016; Mostofa et al., 2018), and therefore can be associated to be present in chloroplast, mitochondria, peroxisomes, cell membranes, nucleus, endoplasmic reticulum, and cytosol.

Ascorbate–glutathione (AsA–GSH) is a key metabolic pathway that keeps the oxidative stress of plants in check through enzymatic [catalase (CAT) EC 1.11.1.6, ascorbate peroxidase (APX) EC 1.11.1.11, dehydroascorbate reductase (DHAR) EC 1.8.5.1, monodehydroascorbate reductase (MDHAR) EC 1.6.5.4, glutathione-*S*-transferase (GST) EC 2.5.1.18, glutathione reductase (GR) EC 1.6.4.2, guaiacol peroxidase (GPX) EC 1.11.1.7, glutathione peroxidase (GPX) EC 1.11.1.9) and non-enzymatic (ascorbic acid (AsA), glutathione (GSH)] antioxidant players (Acosta-Motos et al., 2017; Hasanuzzaman et al., 2020). Similarly, RCS scavenging system also comprises of enzymatic and non-enzymatic compounds (Mano et al., 2019). A rise in MG levels is usually observed under salt stress that triggers the synthesis of glyoxalase: enzyme responsible for the detoxification of MG (Kaur et al., 2016). Under salt stress, mitochondria and chloroplast are specifically found to be affected. Consequently, electron transport chain is disrupted due to stomatal closure. Accordingly, the final electron acceptor in PSI (NADP^+^) of the electron chain suffers a halt in its regeneration that triggers Mehler Reaction (transfer of electron from ferredoxin to oxygen to form O_2_^–^) (Gururani et al., 2015; Acosta-Motos et al., 2017). This O_2_^–^ is further converted to hydrogen peroxide (H_2_O_2_) and superoxide (O_2_^–^) by superoxide dismutase (SOD). Similarly, O_2_^–^ generation in peroxisomes is modulated by APX and CAT activities. Protein denaturation and other structural damages have been reported previously in salt stressed cells, affecting particularly chloroplast and mitochondria due to the accumulation of H_2_O_2_ and O_2_^–^ radicals. Therefore, in order to protect the photosynthetic machinery from ROS, plants regulate ‘xanthophyll cycle’ in which violaxanthin de-epoxidase converts carotenoid violaxanthin to zeaxanthin. This cycle helps in excessive energy dissipation in the form of heat, constituting the main mechanism of excessive energy dissipation, from PSII through non-photochemical quenching (NPQ). Zeaxanthin serves as an antioxidant for photoinhibition and photo-oxidation by scavenging ROS in thylakoid membranes. Similarly, salinity also results in the decrease in chlorophyl content that causes an increase in the anthocyanin and carotenoid accumulation, which help in toxic radicals scavenging and chloroplasts protection from photoinhibition and photooxidation. In the same way, another adapted mechanism for salinity tolerance is photorespiration that constantly recycles carbon dioxide from the decarboxylation of glycine in the mitochondria, so that the Calvin cycle is kept operational. Consequently, it diminishes ROS generation in electron transport chain. In addition, plants also use the water-water cycle to scavenge the ROS and dissipate excessive energy. In this cycle, water generated electrons in PSII are used to; (a) photo-reduce the dioxygen to superoxide in PSI, and (b) recycle ascorbate; thereby sustaining a linear electron flow for ATP generation. Furthermore, NO is involved in the glutathione metabolism by regulating GSH-dependent enzymes, i.e., GST, GR, and GSH. Also, it is reported that NO is a multifunctional molecule that regulates salt stress through genetic and molecular level regulations. Besides the aforementioned mechanisms, plants can also mitigate the oxidative stress through selective up-regulation of antioxidant enzymes, as found in *Lycopersicon pennellii* (Hernandez et al., 1995; Asada, 1999; Hernández et al., 2001; Mittova et al., 2003; Hörtensteiner, 2006; Garriga et al., 2014; Corpas, 2016; Acosta-Motos et al., 2017; Mano et al., 2019).

### Phytohormonal Adjustments

Phytohormones modulate salinity by participating in signaling pathways and gene regulation. Abscisic acid (ABA) is known to regulate genes responsible for stomatal closure and osmoprotectant biosynthesis. It also helps in plant acclimation and inhibition of lateral root growth (Arif et al., 2020). Indole-3-acetic acid (IAA) promotes ion homeostasis by upregulating the expression of various genes including auxin/indoleacetic acid (Aux/IAA), small auxin-up RNA (SAUR) and GH3 (Sun et al., 2018), apart from regulating plant growth and development. Likewise, brassinosteroids (BRs) help plant to cope up with salinity by playing their role in the pollen tube growth, reproduction, proton pump activation, vascular differentiation, photosynthesis, and by improving antioxidant and osmoprotectant contents (Nolan et al., 2020). Also, cytokinins (CKs) are involved in salinity mitigation by increasing shoot to root ratio and antioxidants gene expression (Arif et al., 2020). Furthermore, ethylene is involved in salinity signaling perception and upregulating the expression of osmoprotectant genes, e.g., GB (Fahad et al., 2015). As well, gibberellins (GAs) are increased under salinity and modulate it by improving redox metabolism, sugar signaling, and osmolyte production (Fahad et al., 2015; Rao et al., 2016). Additionally, jasmonic acid (JA) reinforces the expression of arginine decarboxylase, invertase, and Rubisco genes to mitigate salinity. It is also involved in the metabolism of fatty acid along with methyl jasmonate (MeJA). The upregulation of arginine decarboxylase genes results in the modulation of polyamines biosynthesis that serve as osmolytes. Furthermore, it facilitates protein synthesis and CO_2_ fixation under saline conditions (Arif et al., 2020). Additionally, higher amounts of polyamines (PAs), nitrogen-containing aliphatic compounds, are accumulated in salt stressed cells to modulate signaling, cell proliferation, genetic expression, cell turgidity, and senescence (Ismail and Horie, 2017). In addition, salicylic acid (SA) modulates plant salinity to a great deal by participating in signaling pathways and regulating various genes expression. It also modulates ion homeostasis, i.e., limits Na^+^ influx in roots, Na^+^ regulates sequestration and exclusion, facilitates root H^+^-ATPase activity, and augments K^+^ concentration in aerial parts. As well, it upregulates genes of various ion channels to avoid K^+^ leakage (Arif et al., 2020).

### Genomic Adjustments

Plants affected by salinity undertake various genomic adjustments in which various genes are up- and down-regulated. Currently, several advanced genomic techniques have made it possible to assess the molecular changes going-on in a plant under salt stress. Although, this is a set of complex mechanisms that range from transcription to post-translational modifications. Such genetic variation of expression results in the higher production of RNAs and proteins necessary to mitigate salinity. For instance, an upregulation of genes responsible for osmoprotectants biosynthesis is certainly a desired behavior for combating salinity (Amirbakhtiar et al., 2019). Nevertheless, an upregulation of genes is not always the case, rather the genomic behavior of plants may result in down-regulation, moderate expression, or even no expression. Similarly, gene expression can also be altered by the involvement of transcription factors or interfering RNAs. It has been discovered that the endogenous small interfering RNAs (siRNAs) and microRNAs (miRNAs) e.g., miR530a, miR1445, miR1446a-e, miR1447, miR396, miR394, miR393, miR319, miR171 miR169, miR168, etc., also play important roles in stress mitigation (Mangrauthia et al., 2013; Rao et al., 2016).

Following four functional categories of stress-responsive genes of plants have been established by Rao et al. (2016):

(a) Molecular chaperones (e.g., *HSP* genes).
(b) Ion transport or homeostasis (e.g., *SOS* genes, *AtNHX*_1_, and *H^+^-ATPase*).
(c) Dehydration-related transcription factors (e.g., *DREB*).
(d) Senescence-associated genes (e.g., *SAG*).

Some representative and differentially expressed genes (DEGs) are presented in Table 1, where genes or their families are grouped based on their function and involvement in signaling transduction, stress (ionic, osmotic, and oxidative), and metabolites biosynthesis. Generally, all these sets of genes are upregulated under saline environment with few exceptions (Arif et al., 2020). In a recent study, around 5128 DEGs for *Triticum aestivum*, treated with 150 mM NaCl, have been reported (Amirbakhtiar et al., 2019). This huge number of transcripts indicate the level of complexity involved in the regulation of salt stress, although it varies from species to species. Moreover, this study also underlined the upregulation of a set of genes involved particularly in signaling pathways, ion transporters, and oxidative stress.

**TABLE 1:** Representative salt stress regulating genes and their respective functions in plants.

Gene/gene family	Function	References
**Signaling transduction pathways**		
*SOS1*, *SOS2*, *SOS3*, *AtNHX1*	Vacuolar Na^+^/K^+^ antiporter, plasma membrane Na^+^/K^+^ antiporter, protein kinase, Calcium-binding protein.	Sottosanto et al., 2007; Chakraborty et al., 2012; Ismail and Horie, 2017
*ANN4, ACA7, NCL2*, and *GLR*	Ca^2+^ transporters: adjust Ca^2+^ cytosolic concentrations	Amirbakhtiar et al., 2019
*CaM, CIPK*, and *CPK*	Ca^2+^ signaling pathway	Amirbakhtiar et al., 2019
*HAK25, ABAC15, SOS3/CBL4*	Ion homeostasis, and coding for calcium sensing molecules	Fahad et al., 2015; Amirbakhtiar et al., 2019
*GmSALT3*	Encodes various membrane transporters	Yousefirad et al., 2020
*CaM1, CML37, CML27, CML29*, and *CDPK1*	Responsible for the activation of kinase and Ca^2+^ pathway	Amirbakhtiar et al., 2019; Arif et al., 2020
**Ionic, osmotic, and oxidative stress**		
*MAPKKKA, MAPKKK2*, and *MAPKKK3*	Involved in ion homeostasis	Amirbakhtiar et al., 2019; Arif et al., 2020
*MYB, NAC, bHLH, WRKY, bZIP,s* and *AP2/ERF*	Regulate the expression of the genes engaged in dealing with osmotic, ionic, and oxidative stresses arising from salinity	Deinlein et al., 2014; Amirbakhtiar et al., 2019
*TP4-1-like* and *NIP1-1-like; Wrab18, LEA1, LEA3, LEA-D34-Like*, and *LEA14-A; DHN3, DHN4, DHN7*, and *DHN9; P5CS*, and *P5CS*	Genes coding for aquaporins, LEA proteins, dehydrins, and proline synthesis. Involved in plant metabolic pathway	Amirbakhtiar et al., 2019
*CAT, GRXC1, GST, CCOMT, SAM, GAPDH*, and *LAX AP2/ERF*	Mediating in oxidative stress	Amirbakhtiar et al., 2019
*GmLAX3* and *GmST1*	Improve salt tolerance by promoting antioxidant machinery and scavenging ROS	Khan et al., 2019
*MPK3* and *MEKK2*	Ion homeostasis	Amirbakhtiar et al., 2019; Arif et al., 2020
*SUS1, TPS*, and *TPP*	Involved in plant metabolic pathway	Ghaffari et al., 2016
*OsHsp17.0, OsHSP23.7, OsHSP71.1*, and *OsHSP80.2*	Heat-shock proteins, molecular chaperones, proteins transportation	Zou et al., 2009
*GAPDH*	Participates in the glycolytic cycle	Albaladejo et al., 2018
*OSCA1*	Acts as a putative osmosensor	Zörb et al., 2019
**Plant growth and development**		
*CRR-RLK, LRR-RLK, CRR-RLK, PERK*, and *SRK*	Modulates plant growth, development, yield, and stabilizes the cell membrane under salinity	Amirbakhtiar et al., 2019; Guo et al., 2019; Zörb et al., 2019
*AtSTO1*	Biomass, photosynthesis, and pith size	Michler, 2014
*SWEET15*	Modulates vacuolar storage and transport of sugar	Jithesh et al., 2019
*LAX*	Increases vascular development, xylem differentiation, and plant growth	Khan et al., 2019
*RCA1* and *AOX1A*	Promote photosynthetic efficiency	Albaladejo et al., 2018
*CYP94 (cytochrome P450)*	Enhanced expression of *CYP94C2b*	Mochida and Shinozaki, 2011
*CCOMT* and *SAM*	Involved in suberin and lignin biosynthesis	Arif et al., 2020
*OsRab7*	Seedling growth and increased proline content	Dhanaraj et al., 2015

Furthermore, it has been reported that genes coding for calcium sensing molecules (SOS3/CBL4) are up regulated in saline conditions. Their activation leads to the formation of a protein complex resulting in the transcription of Na^+^ antiporter gene (SOS1). Where Ca^2+^ causes Na^+^/H^+^ EXCHANGER 1 (*NHX1*) antiporter assisted Na^+^ sequestration into the vacuole, *SOS1* gene induces Na^+^ efflux from cytosol (Arif et al., 2020). Likewise, genes encoding for proteins of photosynthetic machinery, ROS scavenging activity, SOD, cytochrome production, and isoflavone reductase production have also been found to be upregulated (Fahad et al., 2015; Rao et al., 2016; Amirbakhtiar et al., 2019; Khan et al., 2019). Briefly, all of the enzymes, proteins, osmoprotectants, co-factors, transporters, metabolites, etc. involved in ionic, oxidative, and osmotic stress (as described above: in morphological adjustments and metabolic adjustments) get their respective genes upregulated under the salt stress. For example, calcium pathways and *SOS* signaling genes have been reported to play their role in cell homeostasis and salt acclimation (Fahad et al., 2015). Although, several transcription factors play their intermediate role in these processes and post-translational modifications, but a comprehensive elucidation of their role remains ambiguous.

## Salinity Mitigation Through Conventional Methods

Salts accumulation around the plants root cause salinity in plants. In order to remove these toxic concentrations of salts so as to gain maximum plant yield, commonly implied strategies include flushing, scraping, and leaching. Of these leaching is most widely used strategy, in which irrigation is sustained over the evapotranspiration rates. The excessive amount of water retains the concentrations of salts below their critical limits. For instance, in a study 20–30% extra irrigated water was found to leach 70% of salts from the maize roots (Plaut et al., 2013; Rao et al., 2016). Various irrigation models (e.g., SALTMED, CWPF, Enviro-Gro, WATSUIT, TETRANS, UNSATCHEM, MOPECO-Salt, etc.) based on the leaching principle have also been suggested as salinity mitigation approach and to improve crop yield (Plaut et al., 2013). But these models either considered the salt concentration as constant for any given time or were limited in their performance under various environmental factors; thereby constraining crop yield.

Soil mulching is another conventional approach for salinity mitigation. For this, soil surface is covered with mulch or plastic sheet to enhance water availability by limiting water evaporation. Previous studies conducted on cotton plants have demonstrated positive impact of mulching against salinity in terms of reduced activity of MDA, decreased accumulation of Na^+^ in leaves and roots, inhibition of lipid oxidation, improved photosynthesis, and higher biomass (Dong et al., 2008, 2009). However, this approach has short term effects and is only efficient to affect the upper soil layer. Irrigation water treatment through aeration and/or magnetic processing is another salinity mitigation technique (Plaut et al., 2013). Nevertheless, this is not widely adopted technique due to associated costs and intricacy of process. Another conventional approach is the cultivation of halophytes in saline soils for eliminating or reducing the accumulated salts to the threshold levels for glycophytes. Some halophytes are reported to have salt glands for this purpose that possess the ability to exclude salts, whereas others are reported to have salt hairs that serve to accumulate salts. Additionally, better agronomic and farm management practices can also improve salinity. For instance, with the drip irrigation a controlled amount of water can be applied to the soil, whereby limiting the soil salination. Also, surface and sprinkler irrigations might prove effective to leach down the excessive salts from root zone. Similarly, crop rotation with perennial crops can be practiced particularly in rain-fed areas. Deep roots of perennial crops might help restore the salt-water equilibrium in the soil (Plaut et al., 2013; Rao et al., 2016).

Selection, conventional breeding, and/or genetic engineering for salinity tolerant crops are also reported as salinity mitigation techniques (Athar and Ashraf, 2009; Plaut et al., 2013). In this regard, halophytes or salinity tolerant genotypes could be bred with desired salinity susceptible crop plants to get salt tolerant progeny. Similarly, salinity susceptible plants can be genetically transformed with salt tolerant genes or can be engineered for having salt glands/hairs. Equally, elicitation of plant bioregulators, osmolytes, antioxidants, or other metabolites biosynthesis has also been regarded as a valuable approach (Ashraf and Akram, 2009; Zulfiqar and Ashraf, 2021a). However, despite of the remarkable potential, these strategies are rather limited due to huge amounts of time required and the associated costs. Equally, salinity tolerance is a complex process that is regulated by a large number of genes that obscures the crop breeding and genetic transformation processes. Therefore, attaining a salt resistant transgenic line with its subsequent adoption in field conditions still remains a challenge.

In the same way, use of microbial inoculants, chemical and organic soil amendments, and electro remediation are other promising salinity mitigation strategies that are gaining increased scientific attention lately (Sahab et al., 2020). As chemical soil amendments pose threats to soil microbiota and indirectly to human life, therefore this cannot be regarded as a sustainable approach. An alternative method of salinity mitigation is the exogenous application of nutrients and metabolites that relieves plants from Na^+^ and Cl^–^ injury (Munns, 2002; Plaut et al., 2013). Use of natural PEs, in this regard, can be associated with this strategy of salinity mitigation, although PEs do not only have phytonutrients but also other stress relieving metabolites, e.g., GB, proline, melatonin, etc.

## Use of Plant Extracts Against Salinity

Use of PEs to mitigate salinity can be regarded as an environment friendly and sustainable way of fighting abiotic stress, as it contains no synthetic chemicals. Depending upon the parts of plants used to prepare PE, it may contain various amounts of bioactive compounds (flavonols, phenolics, betaines, amino-polysaccharides, sterols, glucosinolates, terpenoids, furostanol glycosides, etc.), phytohormones, mineral elements, photosynthetic pigments, amino acids, nucleotides or nucleosides, lipids, etc. Due to the associated detoxifying and ROS quenching capabilities of these compounds, PEs are frequently used in pharmacological industry for providing protection against neurodegeneration, diabetes, muscular dystrophy, and cancer like chronic diseases (Pehlivan, 2018). Since these natural compounds are also effective in preventing macromolecules like lipids, proteins, and DNA from damage in animal cells (Pehlivan, 2018), therefore it can be deduced that they might also be effective in plants against salinity as it disrupts redox balance in plants. This has been demonstrated in previous studies that PEs contribute to a better growth, development, yield, disease-, and stress-resistance in plants given the presence of aforementioned compounds (Howladar, 2014; Drobek et al., 2019; Desoky et al., 2020; Zulfiqar et al., 2020b). Nevertheless, further scientific evidences are yet to be excavated to ensure that such a wide variety of molecules in PEs is functional or not. Similarly, viability and quality of PEs is also an aspiring research area.

Sources of PEs, methods of application, and their implications against salinity are discussed below.

### Sources, Preparation, and Application Methods

Plant extracts or botanicals are prepared from natural resources like higher plants. They can be prepared either from a whole plant or from any specific part of the plant, i.e., fruits, flowers, bark, roots, leaves, stems, seeds, pollens, grains, etc. (Semida and Rady, 2014; Lorenzo et al., 2019; Nessim and Kasim, 2019; Rady et al., 2019a; Zulfiqar et al., 2020b; Suryaman et al., 2021). Whereas plant derived products like protein hydrolyzates, polyamines, polyols, amides, etc. fall under the category of plant derived biostimulants (PDBs), as PEs are multicomponent mixtures. However, the extraction of a particular compound or a mixture of compounds can be reinforced by selecting an appropriate method of extract preparation.

**FIGURE 3:**
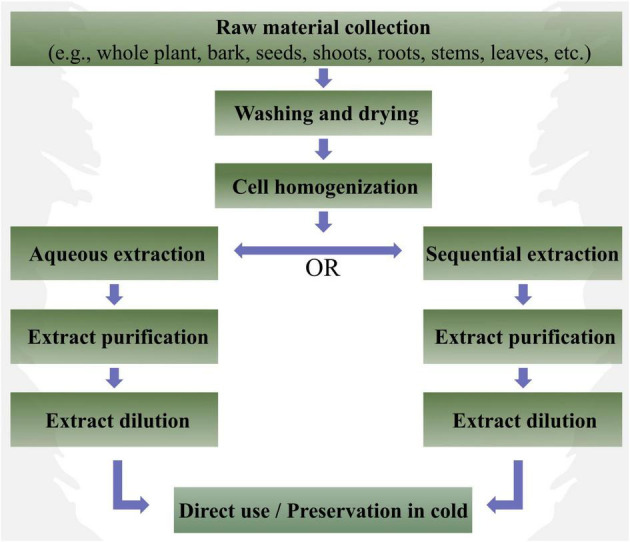
A stepwise illustration of the preparation of plant extracts (PEs).

Conventionally PEs are prepared by maceration. The extraction is done in some solvent either hydrous or organic. For aqueous extraction, desired plant part is macerated or processed mechanically in deionized H_2_O, followed by its purification and centrifugation. The resultant analyte is diluted as per requirement and applied to plant (Rady and Mohamed, 2015; Abd El-Mageed et al., 2017; Yakhin et al., 2017; Ali et al., 2018; Zulfiqar et al., 2020b). In organic solvent extraction, the desired plant part is homogenized in an organic solvent, e.g., ethanol, followed by fractionated extraction with hexane, ethyl acetate, and/or butanol like solvents. Further, the resultant extractants are purified by removing organic solvents through evaporation (Salama et al., 2013; Lim et al., 2014; Brockman and Brennan, 2017; Yakhin et al., 2017; Pehlivan, 2018). Aqueous extraction is considered relatively easier, faster, and economical as compared to the organic solvent-based extraction. Furthermore, several other methods for homogenates preparation can be implied such as bead impact methods, rotor–stator homogenizer, high pressure batch/flow, low-pressure droplet method, ultrasonic processors, etc. (Goldberg, 2008). Besides, these basic methods can be further modified based on the desired extractant, i.e., lipophilic, or hydrophilic. However, an appropriate method of PE preparation is important as it affects the stability characteristics of the formulation (Lötze and Hoffman, 2016). In addition, the extractions carried out using organic solvent, like ethanol, may vary in triggering the physiological response as compared to the extracts prepared through aqueous extraction. The possible reason of such difference could be the variation in physiochemical properties, i.e., pH, temperature, electric charge, surface tension, solubility, etc., of the aqueous and organic extracts. For instance, a subsequent increase in the extract viscosity was observed when pH and temperature were increased (Briceño-Domínguez et al., 2014). Similarly, penetration and assimilation of applied extract may vary depending upon its hydrophilic nature, mode of application, environmental conditions (light, temperature, relative humidity, etc.), ontogenesis, and permeability of plant surface (Fernández and Brown, 2013; Fernández et al., 2017). A turgid cell might not absorb more water resulting in no absorption of aqueous extract. Likewise, a PE prepared through organic solvent-based extraction might also not get absorbed due to the hydrophilic nature of plant cuticle. All these variable factors greatly influence plant physiological response to PEs. A stepwise illustration of the preparation of PEs is presented in Figure 3.

Usually, PEs are applied to plants through following three methods;

(a) Foliar spray (Habib et al., 2012; Lorenzo et al., 2019).
(b) Soil based application (Pehlivan, 2018; Hassanein et al., 2019; Brazales-Cevallos et al., 2022).
(c) Biopriming (seed priming) (Panuccio et al., 2018; ElSayed et al., 2022).

For a good penetration and assimilation of ingredients, PEs should be water soluble (or in any other suitable solvent). To overcome the lipophilicity and molecular size like uptake problems might be solved by mixing PEs with surfactants or other additives (Yakhin et al., 2017). Similarly, the absorbability of PEs also depends upon the molecular structure of cuticle of the plant under study, environmental conditions, and other extrinsic factors (Bulgari et al., 2015).

### Implications of Plant Extracts Against Salinity

The basic aim of the use of PEs is reinforcing the plant responses to salinity so as to sustain the cellular homeostasis. Exogenous application of PEs is found to take part in the signaling, primary and secondary metabolic pathways, and other physiological processes of plant (Table 2). Similarly, morphological, and anatomical adjustments are important mechanisms for stress regulation in plants. Improvement in growth traits including plant height, root and shoot length, fresh and dry weight of shoots, fresh and dry weight of roots, root/shoot ratio, number of leaves, leaf area, leaf thickness leaf relative water content (RWC), number of pods, pods weight, number of seeds, seed weight, grain yield, biological yield, and harvest index results on the use of PEs on salt stressed plants (Habib et al., 2012; Rady et al., 2015; Latif and Mohamed, 2016; Merwad, 2020; Suryaman et al., 2021). These altered characteristics might help glycophytes in better acclimation and tolerance, presumably, by enhanced robustness, a higher accumulation of reserves, photosynthetic pigments, gaseous exchange, and ionic compartmentalization. Nevertheless, nutrients are the fundamental players for such alterations providing energy and substrates. Nutrient uptake is greatly challenged under salinity conditions (Munns and Tester, 2008; Zörb et al., 2019) that can be assuaged by the exogenous application of PEs. Several studies have reported an improvement in nutrient (particularly NPK, Fe, Zn, and Mn) uptake and assimilation in salt stressed plant upon the application of PEs (Bulgari et al., 2015; Rady et al., 2019a; Merwad, 2020).

**TABLE 2:** Use of different plant extracts (PEs) against salinity in various plant species.

Plant extract	Extract type	Application method	Species under study	Salt concentration/ salinity	Results	References
**Seed extracts**						
*Foeniculum vulgare* and *Ammi visnaga*	Seed extracts (2,000 ppm)	Foliar spray	*Vigna unguiculata*	Seawater (EC: 3.5 and 7 dS m^–1^)	Improved growth and yield traits, osmoprotectants content, antioxidant system, RWC, MSI, photosynthetic efficiency, nutrient contents, K^+^/Na^+^ ratio, and anatomical features. Reduced Na^+^ content, EL, and oxidative stress biomarkers.	Desoky et al., 2020
*Garcinia mangostana*	Pericarp extract (1%)	Seed priming	*Vigna radiata* R. Wilczek	0.5 and 1% of NaCl	Increase plant height, leaf area, and yield components.	Suryaman et al., 2021
**Leaf extracts**						
*Moringa oleifera*	Leaf extract (1:30)	Foliar spray	*Phaseolus vulgaris*	200 mM NaCl	Mitigation of oxidative stress and improved morphological and physiological parameters.	Latif and Mohamed, 2016
*Moringa oleifera*	Leaf extract (1:25)	Foliar spray	*Trigonella foenum-graecum*	0, 50, 100 and 200 mM NaCl	Improved ion homeostasis, growth traits, photosynthetic pigments, organic solutes, and total phenols. Increased activities of POD, CAT, APX, and SOD. Identification of new 12 polypeptides.	Latef et al., 2017
*Moringa oleifera*	Leaf extract (3%)	Foliar spray	*Sorghum vulgare* var. sudanense	Non-saline (EC: 3.01 dS m^–1^), medium saline (EC: 6.12 dS m^–1^), highly saline (EC: 12.33 dS m^–1^)	Increased cumulative yield and nutrient uptake.	Merwad, 2017
*Moringa oleifera*	Leaf extract (1:30)	Foliar spray and seed priming	*Helianthus annuus*	Sandy loam (EC: 6.42–6.48 dS m^–1^)	Improved growth traits, RWC, MSI, concentrations of total chlorophylls, total carotenoids, total soluble sugars, free proline and ascorbic acid, ion homeostasis, antioxidant enzymes, seed yield, and seed oil and protein contents.	Taha, 2016
*Moringa oleifera*	Leaf extract (1:30)	Foliar spray and seed priming	*Phaseolus vulgaris*	Saline soil (EC = 6.23–6.28 dS m^–1^)	Improved growth traits, RWC, MSI, concentrations of total chlorophylls, total carotenoids, total soluble sugars, free proline and ascorbic acid, ion homeostasis, antioxidant enzymes, green pods and dry seed yield.	Rady and Mohamed, 2015
*Moringa oleifera*	Leaf extract (1:30)	Foliar spray	*Phaseolus vulgaris*	90 mM NaCl, 1 mM Cd^2+^ (CdCl_2_)	Enhanced growth traits, level of photosynthetic pigments, green pod yield and pod protein, antioxidant enzymes and proline content. No effect on EL and lipid peroxidation	Howladar, 2014
*Ocimum basilicum*	Leaf extract (20%)	Foliar spray	*Vicia faba*	0.0, 50, 100, or 150 mM NaCl	Increased activity of antioxidant enzymes, organic solutes, lipid peroxidation, and ions content	Aboualhamed and Loutfy, 2020
*Moringa oleifera* and *Moringa peregrina*	Leaf extract (2.5, 5, 10, and 20%)	Soil based	*Ocimum basilicum* cv. Cispum	100 mM NaCl	Increased content of proline, MDA, anthocyanin, total carbohydrates, and SOD. Significant increase in growth traits.	Hassanein et al., 2019
*Cupressus macrocarpa*	Leaf Extract (0.5%)	Seed priming	*Cucurbita pepo* cv. Kavili	100 mM NaCl	Enhanced growth, photosynthetic capacity, antioxidant enzyme and rubisco activities, increased contents of AsA, GSH, ratio of K^+^/Na^+^, and proline. Genes upregulation (CuZnSOD2, CAT1, APX, GR, DHAR, and PrxQ)	ElSayed et al., 2022
*Moringa oleifera*	Leaf extract (3%)	Seed priming and foliar spray	*Triticum aestivum* Cv. Sakha 93	Saline soil (EC: 9.10 dS m^–1^)	Enhanced osmotic stress tolerance by stabilizing membrane integrity and decreasing EL. Improved endogenous GSH, AsA, photosynthetic efficiency, photosynthetic pigments, growth traits, ionic- and hormonal-homeostasis.	ur Rehman et al., 2021
*Moringa oleifera*	Leaf extract (1:30)	Foliar spray	*Rosa damascena* var. trigintipetala Dieck	200 mM NaCl	Enhanced growth attributes, chlorophyll content, RWC, proline content, and MSI. Increased radical scavenging activity, total phenols, ratio of K^+^/Na^+^, and antioxidant enzyme activity.	Hassan et al., 2020
*Moringa oleifera*	Leaf extract (–)	Seed priming	*Phaseolus vulgaris* cv. Bronco	100 mM NaCl	Improved growth, yield, content of osmoprotectants, activity of enzymatic and non-enzymatic antioxidants and ratio of K^+^/Na^+^	Rady et al., 2013
*Moringa oleifera*	Leaf extract (6%)	Seed priming	*Triticum aestivum* cultivar Giza 168	120 mM NaCl	Significant amelioration on biomass, yield, osmoprotectants and antioxidant systems	Rady et al., 2019b
*Typha angustifolia*	Leaf extract (4%)	Seed priming	*Pisum sativum* var. Lincoln	120, 240, and 320 mM NaCl	Membrane integrity, increased values of osmotica (proline, total soluble sugars, K^+^, and P), chlorophyll and carotenoid content, and lower EL.	Ghezal et al., 2016
*Rosmarinus officinalis* and *Artemisia herba-alba*	Leaf extracts (1:5)	Seed priming	*Zea mays*	100 mM NaCl	Increased germination percentage and germination indexes, ion compartmentalization of cations and anions, root/shoot ration photosynthetic pigments, and antioxidant system.	Panuccio et al., 2018
*Moringa oleifera*	Leaf extract (1, 3, and 10%)	Seed priming and soil based (irrigation)	*Arabidopsis thaliana*	100 mM NaCl	Activation of ABA-, SA-, AUX-, and ET-related signaling pathways.	Brazales-Cevallos et al., 2022
**Root extracts**						
*Glycyrrhiza glabra*	Root extract (0.5%)	Seed priming	*Pisum sativum* cv. Master-B	150 mM NaCl	Enhanced seedling growth, photosynthetic attributes, AsA, GSH, proline, soluble sugars, α-tocopherols, ratio of K^+^/Na^+^, and antioxidant enzyme activities. Upregulation of CAT-, SOD-, APX-, GR-, DHAR-, and P_rx_Q-encoding genes.	Desoky et al., 2019
*Beta vulgaris*	Root extract (50 mmol Kg^–1^ of GB)	Foliar spray	*Abelmoschus esculentus* cv. Arka-anamika and Sabaz-pari	100 mM NaCl	Improved biomass production, plant yield, various gas exchange characteristics, and leaf ion homeostasis (K^+^, Ca^2+^, Cl^–^, Na^+^, K^+^/Na^+^ ratio in shoot and root).	Habib et al., 2012
*Glycyrrhiza glabra* and *Moringa oleifera*	Licorice: root extract (0.5%); Moringa: leaf extracts (3%)	Foliar spray	*Triticum aestivum* cv. Sakha 93	Saline soil (EC: 9.12 dS m^–1^)	Increased yield, protein content, photosynthetic pigments, and nutrient uptake (NPK, Fe, Zn, and Mn).	Merwad, 2020
*Beta vulgaris*	Root extract (50 mmol Kg^–1^ of GB)	Foliar spray	*Solanum melongena* cv. Dilnasheen and Bemisal	100 mM NaCl	Improved growth, yield, photosynthetic rate, transpiration, stomatal conductance, GB accumulation, and leaf K^+^, Ca^+^, Cl^–^, and Na^+^ content.	Abbas et al., 2010
*Daucus carota*	Root extract (20%)	Seed priming	*Lupinus termis* cv. Gemmeza R_2_	150 mM NaCl	Enhanced growth traits, leaf water content and photosynthetic pigments, total soluble sugars, proteins, alkaloids, MDA, CAT, peroxidase activities and ascorbate content. Preserved cell wall, integrity of chloroplast membranes, normal grana organization and nuclear structure with well-defined nucleoli.	Nessim and Kasim, 2019
*Daucus carota*	Root extract (2%)	Seed priming	*Zea mays*	Seawater induced (Na^+^: 10 mg L^–1^; Cl^–^: 784 mg L^–1^)	Improved growth traits, protection of the photosynthetic pigments, chlorophylls, carotenoids, ion homeostasis, osmolytes, and ROS mitigation.	Latef et al., 2019
*Glycyrrhiza glabra*	Root extract (0.5%)	Seed priming	*Phaseolus vulgaris* cv. Bronco	Saline soil (EC = 7.2 dS m^–1^)	Increased plant growth, yield, photosynthetic pigments, free proline, total soluble carbohydrates, total soluble sugars TSS, nutrients, and selenium, ion homeostasis, RWC, MSI, activities of all enzymatic antioxidants, and anatomical features. Decreased EL, MDA, and ROS content.	Rady et al., 2019a
**Fruit and grains extracts**						
*Zea mays*	Grains extract (6%)	Seed priming and foliar spray	*Phaseolus vulgaris* cv. Paulista	Saline soils (EC = 7.43–7.51 dS m^–1^)	Improved growth and yield components, RWC, MSI, photosynthetic pigments, soluble sugars, proline, N, P, K^+^, Ca^2+^, IAA, GA, and CKs concentrations; K^+^/Na^+^ and Ca^2+^/Na^+^ ratios; SOD, and CAT activities; GSH and AsA contents.	Rady et al., 2019b
*Vaccinium arctostaphylos*	Fruit extract (6%)	Soil based (irrigation)	*Zea mays* Samada 07	200 mM NaCl	Reduced pigment loss, biomass loss, damage to roots and shoots, lipid oxidation, proline synthesis and endogenous H_2_O_2_ concentrations. Improved growth, and levels of antioxidant enzymes.	Pehlivan, 2018
**Bark extracts**						
*Acacia dealbata*	Bark extract (0, 450, or 900 ppm)	Foliar spray	*Allium cepa*	60 and 120 mM NaCl	Attenuation of salinity by increased height, leaf-, root-, total biomass, sugar, and protein content.	Lorenzo et al., 2019
*Salix babylonica*	Bark extract (2, 4%) and leaf extracts (2, 4%)	Seed priming	*Zea mays*	100 mM NaCl	Increased growth traits (shoot fresh weight, root area, etc.), leaf protein concentration. Reduced lipid peroxidation and specific activities of antioxidative enzymes.	Mutlu-Durak and Yildiz Kutman, 2021
**Whole plant extracts**						
*Sorghum bicolor*	Whole plant extract (5%)	Seed priming	*Camelina sativa*	Saline soil (EC: 10 dS m^–1^)	Improved growth traits (emergence percentage, root length, shoot length etc.), α-amylase activity, chlorophyll content, antioxidant enzymes activity and shoot K^+^ ion. Reduced concentrations of H_2_O_2_, MDA, and shoot Na^+^ ion.	Huang et al., 2021
*Rosmarinus officinalis*	Whole plant extract (10 and 20%)	–	*Malus domestica* (seedlings)	50 and 100 mM NaCl	Increased concentrations of ascorbic acid, phenols, trehalose and flavonoids.	Mahmoudand and Dahab, 2018
*Sorghum bicolor*	Whole plant extract (5%)	Seed priming	*Triticum aestivum*	Saline soil (EC: 4 and 10 dS m^–1^)	Increased total phenolics, total soluble sugars, proteins, α-amylase activity, chlorophyll contents, and K^+^ ions. Decreased Na^+^ content.	Bajwa et al., 2018

*ABA, abscisic acid; AsA, ascorbic acid; APX, ascorbate peroxidase; AUX, auxin; CAT, catalase; CKs, cytokinins; DHAR, dehydroascorbate reductase; EL, electrolyte leakage; ET, ethylene; GA, gibberellic acid; GSH, Glutathione; GR, glutathione reductase; H_2_O_2_, hydrogen peroxide; IAA, indole-3-acetic acid; MDA, malondialdehyde; MSI, membrane stability index; POD, guaiacol peroxidase; P_rx_Q, peroxiredoxins; ROS, reactive oxygen species; Rubisco, ribulose-1,5-bisphosphate carboxylase/oxygenase; RWC, relative water content; SOD, superoxide dismutase; SA, salicylic acid.*

The PEs rich in antioxidants can be associated to facilitate the stress mitigation processes through enzymatic and non-enzymatic processes. For instance, salinity caused osmotic stress results in lower ψ_s_, photosynthetic performance (*F*_v_/*F*_m_, intracellular CO_2_ concentration, CO_2_ assimilation rate, net photosynthetic rate, transpiration rate, and stomatal conductance), altered ions concentrations (K^+^/Na^+^, Ca^2+^/Na^+^, K^+^ + Ca^2+^/Na^+^), and disrupted hormonal content. These perturbations could be mitigated by the enhanced content of GSH and AsA that improve the tolerance by decreasing electrolyte leakage and stabilizing membrane integrity. Enhanced content of osmoprotectants/osmolytes (GB, proline, pipecolate betaine mannitol, sorbitol, etc.) could be attributed to enhanced ionic, hormonal, and osmotic adjustments that result in improved acclimation, photosynthetic efficiency, growth, and yield (Bulgari et al., 2015; ur Rehman et al., 2021). Overall, lower concentrations of PEs have been found to induce these positive results as higher concentrations might produce harmful results.

**FIGURE 4:**
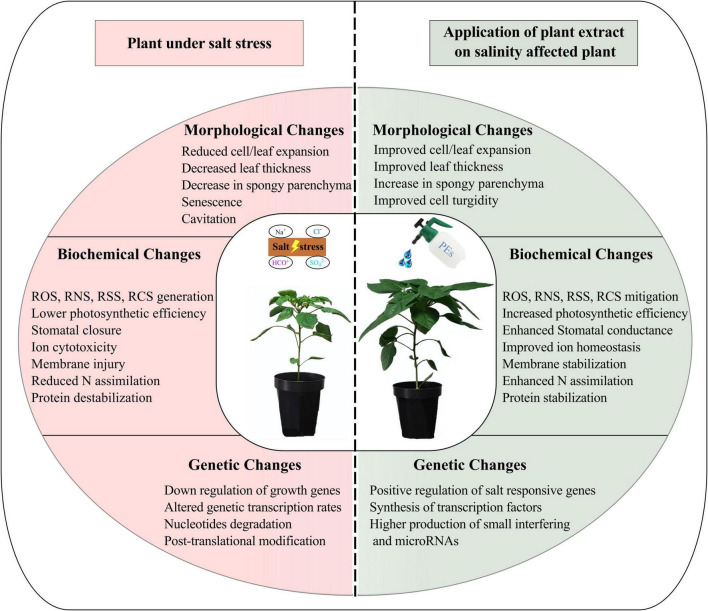
Illustration of the impact of salinity, in terms of morphological, biochemical, and genetic changes, on plants **(Left)** in comparison with the plants supplied with PEs **(Right)**.

Similarly, PEs are efficient sources of ROS and RNS scavengers that are needed to mitigate toxic radicals and stabilize cell homeostasis. They can restore the K^+^/Na^+^ ratios of both root and leaf (Abbas et al., 2010). Desoky et al. (2020) reported a decreased in the contents of these reactive species on the application of seed extracts on *Vigna unguiculata* irrigated with seawater (EC: 3.5 and 7 dS m^–1^). Among others, they attributed the improved K^+^/Na^+^ ratio and membrane integrity to antioxidants and polyphenols rich seed extracts. Analogous results have been reported in case of *Phaseolus vulgaris, Vicia faba, Triticum aestivum, Lupinus termis*, and *Zea mays* plants subjected to varying degree of salt concentrations (Rady and Mohamed, 2015; Latif and Mohamed, 2016; Latef et al., 2019; Nessim and Kasim, 2019; Aboualhamed and Loutfy, 2020; ur Rehman et al., 2021). In another study, presence of phenols, flavonoids, and AsA in *Rosmarinus officinalis* L. extracts was associated to salinity alleviation in apple seedlings (Mahmoudand and Dahab, 2018). Each plant responds differently as per PEs used and therefore various oxidative stress mitigation strategies can be observed. Likewise, the negligible or no activity of some antioxidants might be attributed to various fluctuations in the activation of corresponding transcription factor/genes. A demonstration of negative effects of salinity on plant and its mitigation by PEs is illustrated in Figure 4.

In the same way, PEs carried phytohormones, particularly ABA, JA, ethylene, and SA, stimulate the signaling pathway; whereby triggering various transcription factors and stress related genes. Equally, phytohormones might also be associated to bolster the photosynthetic machinery and overall ionic balance in the cell. In a recent study, *Cupressus macrocarpa* foliar extract primed seed were found to have upregulated various stress related genes (*CuZnSOD2*, *CAT1*, *DHAR, APX*, *P_*rx*_Q*, and *GR*) in *Cucurbita pepo* (ElSayed et al., 2022). Upregulation of *P_*rx*_Q*-, *APX*-, *SOD*-, *DHAR*-, *GR*-, and *CAT*-encoding genes have also reported in *Pisum sativum* when its seeds were primed with *Glycyrrhiza glabra* root extracts and subjected to 150 mM NaCl stress (Desoky et al., 2019). Another evidence of the possible role of PEs in signaling was backed in a study on *Arabidopsis*, where moringa leaf extracts facilitated salinity mitigation by transcriptionally activating ABA-, SA-, AUX-, and ET-related signaling pathways (Brazales-Cevallos et al., 2022). ABA is also reported to regulate the transcription factor *ABI5* (ABSCISIC ACID INSENSIVE 1) that is required by plants to activate *ABI5* expression and salt acclimation (Zörb et al., 2019). However, comprehensive studies to establish the molecular basis of PEs phytohormones in signaling are either in dearth or non-existent.

## Limitations and Future Perspective

Plant extracts being amalgams of various biological compounds make it difficult to map out their exact mode of action. Studies undertaken to compare the individual effects of various osmolytes (e.g., GB) or phytohormones (e.g., SA) along with PEs, concluded the supremacy of PEs as being more efficient (Abbas et al., 2010; Rady et al., 2015). Similarly, the role of PEs in signal transduction remains ambiguous as exogenous application of relevant compounds (i.e., ethylene, AsA, etc.) can also elicit the plant response. Either PEs work as elicitors of natural compounds or PEs carried molecule assimilation results in the desired results, needs further investigation. Similarly, the involvement of protein kinases has already been documented in saline environments (Zörb et al., 2019; Arif et al., 2020), but no such studies have been undertaken upon the use of PEs. Likewise, elucidation of the potential role of reactive sulfur species (RSS) (Corpas and Barroso, 2015) and RCS with respect to salinity and its subsequent mitigation through PEs might open new avenues of research. In addition, application of nanoparticles (NPs) coated PEs might improve their efficiency exponentially as various NPs have been documented effective against salinity (Zulfiqar and Ashraf, 2021b). Nevertheless, intensive research is needed for the application of PEs coated with NPs because of the reported toxicities of NPs based on their physiochemical properties and plant species (Ahmad et al., 2021a).

The role of PEs in morphological and anatomical traits like shape and size of palisade and mesophyll cells, integrity of grana and thylakoids, integrity of cristae, the number and size of plastoglobuli, number and diameter of xylem and phloem tissues, width of cortex, suberin and casparian strips development, root-, shoot-apex, endodermis, and exodermis etc. needs more scientific attention, as these factors play crucial role in gaseous exchanges, ions permeability, ψ_w_, ψ_s_, and energy generation processes (Acosta-Motos et al., 2017). Analogously, comprehensive studies using omics approaches (genomics, transcriptomics, proteomics, metabolomics, and bioinformatics) can further shed lights on positive or negative regulators of morphological-, metabolic-, and genomic-adjustments, target molecules, and the potential receptors activated by the use of PEs. The already identified signaling signatures, genes, and other key metabolites can be used to investigate such processes on the use of PEs. The influence of PEs on interference RNA mechanism to combat salinity also remains an enticing research area.

Use of PEs to combat salinity is a green, ecofriendly, and sustainable approach. This also opens further doors of investigation on the use of invasive plant species to be used as salinity moderator. Another important aspect is that plant response owing to PEs varies greatly from species to species and even within the same species. This might be answered by undertaking further comparative studies. Furthermore, use of salinity to trigger the production of various osmolytes and antioxidants can be utilized as an elicitation approach. Plants subjected to salinity can be used to prepare PEs that might prove more promising against salinity than conventional ones.

## Conclusion

Salt stress has become a consistent problem in agriculture over the past few years, and was reported to culminate around 900 million ha in 2020. Plants perceive salinity by sensors, e.g., cell surface-based receptors, protein kinases, etc., resulting in a cascade of phosphorylation that regulates subsequent genetic expression. Salinity results in ionic, osmotic, and oxidative stress, which further disrupts various physiological and metabolic processes in plants. Use of PEs to combat salinity is an efficient, economical, and sustainable approach. Whole plants or parts of plants, i.e., roots, leaves, flowers, bark, seeds, pollens, etc. can be used to prepare PEs through aqueous or organic-solvent extraction techniques. PEs are multicomponent organic mixtures, containing vitamins, carotenoids, amino acids, phytohormones, mineral nutrients, phenolics, and antioxidants, etc., which facilitate stress signaling, genes regulation, redox metabolism, and synthesis of various proteins and metabolites. The degree of impact of PEs depends on various factors like plant species, age of plant, application method, etc. Molecular characterization of the PEs produced effects can pave the way for elucidating their comprehensive mechanism of action.

## Author Contributions

AA, BB, and VM: conceptualization. AA: study design, data collection and analysis, draft writing and editing, and Illustrations. VM and BB: critical analysis, revision, and supervision. All authors have read and agreed to the published version of the manuscript.

## Conflict of Interest

The authors declare that the research was conducted in the absence of any commercial or financial relationships that could be construed as a potential conflict of interest.

## Publisher’s Note

All claims expressed in this article are solely those of the authors and do not necessarily represent those of their affiliated organizations, or those of the publisher, the editors and the reviewers. Any product that may be evaluated in this article, or claim that may be made by its manufacturer, is not guaranteed or endorsed by the publisher.
